# Commutators of Singular Integral Operators Satisfying a Variant of a Lipschitz Condition

**DOI:** 10.1155/2014/641705

**Published:** 2014-05-04

**Authors:** Pu Zhang, Daiqing Zhang

**Affiliations:** ^1^Department of Mathematics, Mudanjiang Normal University, Mudanjiang 157011, China; ^2^School of Mathematical Sciences, Xiamen University, Xiamen 361005, China

## Abstract

Let *T* be a singular integral operator with its kernel satisfying |*K*(*x* − *y*) − ∑_*k*=1_^*ℓ*^‍*B*_*k*_(*x*)*ϕ*_*k*_(*y*)| ≤ *C* | *y*|^*γ*^/|*x* − *y*|^*n*+*γ*^, |*x* | > 2 | *y* | > 0, where *B*_*k*_ and *ϕ*_*k*_  (*k* = 1,…, *ℓ*) are appropriate functions and *γ* and *C* are positive constants. For $\vec{b}=(b_{1},\ldots ,b_{m})$ with *b*_*j*_ ∈ *BMO*(ℝ^*n*^), the multilinear commutator $T_{\vec{b}}$ generated by *T* and $\vec{b}$ is formally defined by $T_{\vec{b}}f(x)=\int {\mathbb{R}}^{n}\left[\prod_{j=1}^{m}‍(b_{j}(x)-b_{j}(y))\right]K(x,y)f(y)dy$. In this paper, the weighted *L* ^*p*^-boundedness and the weighted weak type *L*log⁡*L* estimate for the multilinear commutator $T_{\vec{b}}$ are established.

## 1. Introduction and Results

In the classical Calderón-Zygmund theory, the Hörmander's condition

(1) $$\begin{array}{c} \int_{\left|x|>2|y\right|}\left|K\left(x-y\right)-K\left(x\right)\right|dx\leq C, \end{array}$$

introduced by Hörmander [1], plays a fundamental role in the theory of Calderón-Zygmund operators. On the other hand, singular integral operators whose kernels do not satisfy the Hörmander's condition have been extensively studied.

In 1997, in order to study the *L* ^*p*^-boundedness of certain singular integral operators, Grubb and Moore [2] introduced the following variant of the classical Hörmander's condition,

(2) $$\begin{array}{c} \int_{\left|x|>2|y\right|}\left|K\left(x-y\right)-\overset{l}{\underset{k=1}{\sum }}B_{k}\left(x\right)\phi_{k}\left(y\right)\right|dx\leq C, \end{array}$$

where *B*_*k*_ and *ϕ*_*k*_'s are appropriate functions (see Theorem 3 below). As an example we note that the kernel *K*(*x*) = sin*x*/*x* verifies (2), but it is not a Calderón-Zygmund kernel since its derivative does not decay quickly enough at infinity (see [2] or [3]).

Obviously, if we take *l* = 1, *B*_1_(*x*) = *K*(*x*) and *ϕ*_1_(*y*) ≡ 1, then condition (2) is exactly the classical Hörmander's condition (1).

**Definition 1**

We say that a nonnegative locally integrable function *g* defined on *R* ^*n*^ satisfies the reverse Hölder *RH*_*∞*_ condition, in short, *g* ∈ *RH*_*∞*_(*R* ^*n*^), if there is a constant *C* > 0 such that for every cube *Q* ⊂ *R* ^*n*^ centered at the origin we have

(3) $$\begin{array}{c} 0<\underset{x\in Q}{\sup}g\left(x\right)\leq C\frac{1}{\left|Q\right|}\int_{Q}g\left(x\right)dx. \end{array}$$

The smallest constant *C* is said to be the *RH*_*∞*_ constant of *g*.

**Remark 2**

It is easy to see that if *g*(*x*) ∈ *RH*_*∞*_(*R* ^*n*^), then also *g*(−*x*) ∈ *RH*_*∞*_(*R* ^*n*^) (see [3] Remark 2.4).

In [2], Grubb and Moore established the *L* ^*p*^-boundedness and the weak type (1,1) estimates for the singular integral operators with kernels satisfying (2).

It is well known that the classical Hörmander's condition (1) is too weak to get weighted inequalities for the classical Calderón-Zygmund operators by any known method. The usual hypothesis on the kernel *K* to obtain them is the Lipschitz condition

(4) $$\begin{array}{c} \left|K\left(x-y\right)-K\left(x\right)\right|\leq \frac{C{\left|y\right|}^{\gamma }}{{\left|x-y\right|}^{n+\gamma }},\;\left|x\right|>c\left|y\right|. \end{array}$$

Conditions, the so-called *L* ^*r*^-Hörmander's condition, weaker than (4), but stronger than (1), have been also considered in [4, 5] (also see [6, 7]).

In 2003, Trujillo-González [3] establishes the weighted norm inequalities for *T* when *K* satisfies a variant of the Lipschitz condition (see (6) below).

As usual, we denote by *A*_*p*_  (1 ≤ *p* ≤ *∞*) the Muckenhoupt weights classes (see [8], or [9] and [10]). For a weight *ω*, 1 ≤ *p* < *∞* and a measurable set *E*, we write

(5) ||f||Lp(ω)=(∫Rn|f(x)|pω(x)dx)1/p,ω(E)=∫Eω(x)dx.

**Theorem 3 (see [3])**

Let *K* ∈ *L* ^2^(*R* ^*n*^). Suppose that there is a constant *C*_0_ > 0, such that

 (*K*_1_) ${||\hat{K}||}_{\infty }\leq C_{0}$;
 (*K*_2_) |*K*(*x*)|≤*C*_0_ | *x*|^−*n*^;
 (*K*_3_) there exist functions *B*_1_,…, *B*_*l*_ ∈ *L*_loc⁡_^1^(*R* ^*n*^∖{0}) and {*ϕ*_1_,…, *ϕ*_*l*_} ⊂ *L* ^*∞*^(*R* ^*n*^) such that |det⁡[*ϕ*_*k*_(*y*_*i*_)]|^2^ ∈ *RH*_*∞*_(*R* ^*nl*^), where *y*_*i*_ ∈ *R* ^*n*^ and *i*, *k* = 1,…, *l*;
 (*K*_4_) for a fixed *γ* > 0 and for any |*x* | >2 | *y* | >0,
  (6) $$\begin{array}{c} \left|K\left(x-y\right)-\overset{l}{\underset{k=1}{\sum }}B_{k}\left(x\right)\phi_{k}\left(y\right)\right|\leq C_{0}\frac{{\left|y\right|}^{\gamma }}{{\left|x-y\right|}^{n+\gamma }}. \end{array}$$
 For *f* ∈ *C*_0_^*∞*^(*R* ^*n*^), we defined the convolution operator associated to the kernel *K* by
  (7) $$\begin{array}{c} Tf\left(x\right)=\int_{R^{n}}K\left(x-y\right)f\left(y\right)dy. \end{array}$$
(1) Let 1 < *p* < *∞* and *ω* ∈ *A*_*p*_. Then there exists a constant *C* > 0 such that
  (8) $$\begin{array}{c} \int_{R^{n}}{\left|Tf\left(x\right)\right|}^{p}\omega \left(x\right)dx\leq C\int_{R^{n}}{\left|f\left(x\right)\right|}^{p}\omega \left(x\right)dx. \end{array}$$
(2) Let *ω* ∈ *A*_1_. Then there exists a constant *C* > 0 such that for all *λ* > 0
  (9) $$\begin{array}{c} \omega \left(\left\{x\in R^{n}:\left|Tf\left(x\right)\right|>\lambda \right\}\right)\leq \frac{C}{\lambda }\int_{R^{n}}\left|f\left(y\right)\right|\omega \left(y\right)dy. \end{array}$$

It is easy to see that any kernel satisfies condition (6) and also verifies (2). Obviously, if we take *l* = 1, *B*_1_(*x*) = *K*(*x*), and *ϕ*_1_(*y*) ≡ 1, then condition (6) is exactly the classical Lipschitz condition (4). We remark that the function *K*(*x*) = sin*x*/*x* satisfies conditions (*K*_1_)–(*K*_4_), but does not satisfy the Hörmander's condition (1) (see [11] page 5).

Under the assumption of Theorem 3, several authors have studied two-weight inequalities for the convolution operator *T*, for example [11–13]. Recently, the authors [14] introduce a variant of the classical *L* ^*r*^-Hörmander's condition in the scope of (2) and establish the weighted norm inequalities for singular integral operator with its kernel satisfying such a variant of the classical *L* ^*r*^-Hörmander's condition.

On the other hand, the commutators of singular integral operators have been widely studied by many authors; see, for example, [15–22] and the references therein. Given a locally integrable function *b* and a linear operator *T* with kernel *K*, the linear commutator [*b*, *T*] is formally defined by

(10) $$\begin{array}{c} \left[b,T\right]f=bT\left(f\right)-T\left(bf\right). \end{array}$$

For $\vec{b}=(b_{1},\ldots ,b_{m})$ with *b*_*j*_ ∈ *BMO*(*R* ^*n*^)  (*j* = 1,…, *m*). The generalized commutator, the so-called the multilinear commutator, $T_{\vec{b}}$ is formally defined by

(11) $$\begin{array}{c} T_{\vec{b}}f\left(x\right)=\int_{R^{n}}\left[\overset{m}{\underset{i=1}{\prod }}\left(b_{j}\left(x\right)-b_{j}\left(y\right)\right)\right]K\left(x,y\right)f\left(y\right)dy. \end{array}$$

In 2002, Pérez and Trujillo-González [22] studied the sharp weighted estimates for the multilinear commutators of the classical Calderón-Zygmund operators. In 2006, Zhang [23] studied the weighted estimates for maximal multilinear commutators.

In 1993, Alvarez et al. [15] established a generalized boundedness criterion for the commutators of linear operators. Now, we restate Theorem 2.13 in [15] in the following strong form.

**Theorem 4 (see [15])**

Let *K* be a linear operator and 1 < *p* < *∞*. Suppose that for all *ω* ∈ *A*_*p*_(*R* ^*n*^), the linear operator *K* satisfies the following weighted estimate

(12) $$\begin{array}{c} {||Kf||}_{L^{p}(\omega )}\leq C{||f||}_{L^{p}(\omega )}, \end{array}$$

where the constant *C* depends only on *n*, *p*, and the *A*_*p*_ constant of *ω*. Then for *b* ∈ *BMO*(*R* ^*n*^) and any weight function *ν* ∈ *A*_*p*_, the commutator [*b*, *K*] is bounded from *L* ^*p*^(*ν*) to *L* ^*p*^(*ν*) with bound depending on *n*, *p*, and the *A*_*p*_ constant of *ω*.

The goal of this paper is to study the weighted norm inequalities for multilinear commutator of the convolution operator *T* defined by (7) with its kernel satisfying (*K*_1_)–(*K*_4_).

By Theorem 3 and applying Theorem 4 *m*-times, we can easily get the following weighted *L* ^*p*^ inequalities for the multilinear commutator $T_{\vec{b}}$.

**Theorem 5**

Let *T* be the singular integral operator defined by (7) with its kernel satisfying (*K*_1_)–(*K*_4_). If 1 < *p* < *∞*, *ω* ∈ *A*_*p*_, and *b*_*j*_ ∈ *BMO*(*R* ^*n*^)  (*j* = 1,…, *m*), then there exists a positive constant *C* such that

(13) $$\begin{array}{c} \int_{R^{n}}{\left|T_{\vec{b}}f\left(x\right)\right|}^{p}\omega \left(x\right)dx\leq C\int_{R^{n}}{\left|f\left(x\right)\right|}^{p}\omega \left(x\right)dx. \end{array}$$

It is well-known that, in general, the linear commutator of Calderón-Zygmund operator fails to be of weak type (1,1) and does not map *H* ^1^(*R* ^*n*^) into *L* ^1^(*R* ^*n*^) when *b* ∈ *BMO*(*R* ^*n*^); see [20] for more details. Instead, an endpoint theory was provided for this operator, such as the weak type *L*log⁡*L* estimate and the weak type (*H* ^1^, *L* ^1^) estimate (see [20, 24]).

The main result of this paper is the following weak type *L*log⁡*L* estimate for multilinear commutator of the singular integral operator defined in Theorem 3.

**Theorem 6**

Let *T* be the singular integral operator defined by (7) with its kernel satisfying (*K*_1_)–(*K*_4_). If *ω* ∈ *A*_1_ and *b*_*j*_ ∈ *BMO*(*R* ^*n*^)  (*j* = 1,…, *m*), then, for all *λ* > 0,

(14) ω({x∈Rn:|Tb→f(x)|>λ})  ≤C∫Rn|f(y)|λ(1+log⁡+|f(y)|λ)mω(y)dy,

where *C* is a positive constant independent of *λ* and *f*.

Throughout this paper, *γ* denotes the positive number appeared in (6). As usual, the letter *C* stands for a positive constant which is independent of the main parameters and not necessary the same at each occurrence. A cube *Q* in *R* ^*n*^ always means a cube whose sides parallel to the coordinate axes. For a cube *Q* and a number *t* > 0, we denote by *tQ* the cube with the same center and *t*-times the side length as *Q*. The symbol *A* ≈ *B* means there exist positive constants *C*_1_ and *C*_2_ such that *C*_1_ *A* ≤ *B* ≤ *C*_2_ *A*.

This paper is arranged as follows. In Section 2, we formulate some preliminaries and lemmas we need. In Section 3 we will prove Theorem 6 for the case *m* = 1, and in the last section we prove Theorem 6 for the general case *m* > 1.

## 2. Preliminaries and Lemmas

In this section, we give some notations and results needed for the proof of the main result.

### 2.1. Muckenhoupt Weight Classes

A nonnegative locally integrable function defined on *R* ^*n*^ is called a weight. We say a weight ∈*A*_*p*_  (1 < *p* < *∞*), if there exists a constant *C* > 0 such that for all cubes *Q* ⊂ *R* ^*n*^

(15) $$\begin{array}{c} \left(\frac{1}{\left|Q\right|}\int_{Q}\omega \left(x\right)dx\right){\left(\frac{1}{\left|Q\right|}\int_{Q}\omega {\left(x\right)}^{-1/\left(p-1\right)}dx\right)}^{p-1}\leq C. \end{array}$$

We say a weight *ω* ∈ *A*_1_, if there exists a constant *C* > 0 such that for all cubes *Q* ⊂ *R* ^*n*^

(16) $$\begin{array}{c} \frac{1}{\left|Q\right|}\int_{Q}\omega \left(y\right)dy\leq C\underset{y\in Q}{\text{ess}\;\inf}\omega \left(y\right). \end{array}$$

The *A*_*∞*_ weights class is defined by *A*_*∞*_ = ⋃_1<*p*<*∞*_ *A*_*p*_. There is also another characterization of the *A*_*∞*_ class, that is, we say a weight *ω* ∈ *A*_*∞*_, if there exist positive constants *C* and *δ* such that, for any cube *Q* and any measurable set *E* ⊂ *Q*, there exist

(17) $$\begin{array}{c} \frac{\omega \left(E\right)}{\omega \left(Q\right)}\leq C{\left(\frac{\left|E\right|}{\left|Q\right|}\right)}^{\delta }. \end{array}$$

### 2.2. Projection of Function

Now, let us recall the definition of the projection of a function (see [2] or [3]). By the projection of an *L* ^1^-function *f* onto a finite-dimensional subspace *Y* we refer to such an element, if it exists *P*(*f*) of *Y* verifying

(18) $$\begin{array}{c} \overset{}{\underset{}{\int }}f\left(x\right)\bar{h}\left(x\right)dx=\overset{}{\underset{}{\int }}P\left(f\right)\left(x\right)\bar{h}\left(x\right)dx,\;\text{for}\;\;\text{every}\;h\in Y. \end{array}$$

**Lemma 7 (see [2])**

Suppose {*ϕ*_1_,…, *ϕ*_*l*_} is a finite family of bounded functions on *R* ^*n*^ such that |det⁡[*ϕ*_*k*_(*y*_*i*_)]|^2^ ∈ *RH*_*∞*_(*R* ^*nl*^). Then, for any cube *Q* centered at the origin and any *f* ∈ *L* ^1^(*Q*), there exists the projection *P*_*Q*_ *f* of *f* onto span⁡{*ϕ*_1_,…, *ϕ*_*l*_} ⊂ *L* ^1^(*Q*) and satisfies

(19) $$\begin{array}{c} \underset{y\in Q}{\sup}\left|P_{Q}f\left(y\right)\right|\leq C\frac{1}{\left|Q\right|}\int_{Q}\left|f\left(y\right)\right|dy, \end{array}$$

where the constant *C* depends only on *n*, *l*, and the *RH*_*∞*_ constant of |det⁡[*ϕ*_*k*_(*y*_*i*_)]|^2^.

### 2.3. Notations Related to Orlicz Spaces

A function Φ : [0, *∞*)→[0, *∞*) is said to be a Young function, if Φ is continuous, convex, and increasing with Φ(0) = 0 and lim⁡_*t*→*∞*_Φ(*t*) = *∞*. We use $\tilde{\Phi }$ to denote the complementary Young function associated to Φ; that is,

(20) $$\begin{array}{c} \tilde{\Phi }\left(s\right)=\underset{0\leq t<\infty }{\sup}\left\{st-\Phi \left(t\right)\right\},\;0\leq s<\infty . \end{array}$$

The Φ-average of a locally integrable function *f* over a cube *Q* ⊂ *R* ^*n*^ is defined by

(21) $$\begin{array}{c} {||f||}_{\Phi ,\;Q}=\inf\left\{\lambda >0:\frac{1}{\left|Q\right|}\int_{Q}\Phi \left(\frac{\left|f\left(y\right)\right|}{\lambda }\right)dy\leq 1\right\}, \end{array}$$

which satisfies the following inequalities (see [25], p. 69, or formula (7) in [21]):

(22) $$\begin{array}{c} {||f||}_{\Phi ,\;Q}\leq \underset{\eta >0}{\inf}\left\{\eta +\frac{\eta }{\left|Q\right|}\int_{Q}\Phi \left(\frac{\left|f\left(y\right)\right|}{\eta }\right)dy\right\}\leq 2{||f||}_{\Phi ,Q}. \end{array}$$

The Young function that we are going to use is Φ_*α*_(*t*) = *t*(1 + log⁡^+^ *t*)^*α*^  (*α* > 0) with its complementary Young function ${\tilde{\Phi }}_{\alpha }(t)\approx \exp(t^{1/\alpha })$. Denote

(23) $$\begin{array}{c} {||f||}_{L{\left(\log L\right)}^{\alpha },\;Q}={||f||}_{\Phi_{\alpha },\;Q},\;\;{||f||}_{\exp L^{1/\alpha },\;Q}={||f||}_{{\tilde{\Phi }}_{\alpha },\;Q}. \end{array}$$

When *α* = 1, we simply write Φ(*t*) = *t*(1 + log⁡^+^ *t*) and $\tilde{\Phi }(t)\approx e^{t}$, and ||*f*||_*L*(log⁡*L*), *Q*_ = ||*f*||_Φ, *Q*_ and ${||f||}_{\exp L,\;Q}={||f||}_{\tilde{\Phi },\;Q}$.

The following generalized Hölder's inequality holds (see (2.5) in [22]):

(24) 1|Q|∫Q|f1(y)f2(y)⋯fm(y)g(y)|dy  ≤C||g||L(log⁡L)m, Q∏j=1m||fj||exp⁡L, Q.

We also need the following notations (see [26] pages 1712-1713). For *ω* ∈ *A*_*∞*_ and a cube *Q* ⊂ *R* ^*n*^, denote

(25) ||f||L(log⁡L)m, Q, ω =inf⁡{λ>0:1ω(Q)∫QΦm(|f(y)|λ)ω(y)dy≤1},||f||exp⁡L1/m, Q, ω =inf⁡{λ>0:1ω(Q)∫QΦ~m(|f(y)|λ)ω(y)dy≤1}.

Similarly to (22), we have

(26) ||f||L(log⁡L)m, Q, ω ≈inf⁡η>0{η+ηω(Q)∫QΦm(|f(y)|η)ω(y)dy}.

There also holds the following generalized Hölder's inequality:

(27) 1ω(Q)∫Q|f1(y)⋯fm(y)g(y)|ω(y)dy  ≤C||g||L(log⁡L)m, Q, ω∏j=1m||fj||exp⁡L, Q, ω.

### 2.4. Lemmas

The following generalized Young's inequality is from [22] Lemma 8. We note that when *k* = 2, it is proved by O'Neil in [27].

**Lemma 8 (the generalized Young's inequality)**

*φ*_0_, *φ*_1_,…*φ*_*k*_ are real-valued, nonnegative, nondecreasing, left continuous functions defined on [0, *∞*). For 0 ≤ *t* < *∞*, define *φ*_*j*_^−1^(*t*) = inf⁡{*s* : *φ*_*j*_(*s*) > *t*}  (*j* = 0,1,…, *k*). If for all 0 ≤ *t* < *∞*

(28) $$\begin{array}{c} \varphi_{1}^{-1}\left(t\right)\cdots \varphi_{k}^{-1}\left(t\right)\leq \varphi_{0}^{-1}\left(t\right). \end{array}$$

Then, for all 0 ≤ *t*_1_, *t*_2_,…, *t*_*k*_ < *∞*, there exist

(29) $$\begin{array}{c} \varphi_{0}\left(t_{1}t_{2}\cdots t_{k}\right)\leq \varphi_{1}\left(t_{1}\right)+\varphi_{1}\left(t_{2}\right)+\cdots +\varphi_{k}\left(t_{k}\right). \end{array}$$

For Φ_*k*_(*t*) = *t*(1 + log⁡^+^ *t*)^*k*^  (*k* = 1,…, *m*) and Ψ(*t*) = *e* ^*t*^ − 1, we have Φ_*k*_^−1^(*t*) ≈ *t*/(log⁡*t*)^*k*^ and Ψ^−1^(*t*) ≈ log⁡*t* (see [21] page 35). Then for any integer *j* with 1 ≤ *j* ≤ *m* − 1, we have

(30) $$\begin{array}{c} \Phi_{m}^{-1}\left(t\right)\underset{m-j}{\underset{︸}{\Psi^{-1}\left(t\right)\cdots \Psi^{-1}\left(t\right)}}\leq C\Phi_{j}^{-1}\left(t\right):=A^{-1}\left(t\right). \end{array}$$

Noting that *A*(*t*) = Φ_*j*_(*C* ^−1^ *t*) since *A* ^−1^(*t*) = *C*Φ_*j*_^−1^(*t*), then it follows from Lemma 8 that, for all 0 ≤ *s*, *t*_1_, *t*_2_,…, *t*_*m*−*j*_ < *∞*, we have

(31) Φj(C−1s·t1⋯tm−j)=A(s·t1⋯tm−j)≤Φm(s)+Ψ(t1)+⋯+Ψ(tm−j).  

For a locally integrable function *f* and a cube *Q*, denote

(32) $$\begin{array}{c} f_{Q}={\left(f\right)}_{Q}=\frac{1}{\left|Q\right|}\int_{Q}f\left(y\right)dy. \end{array}$$

**Lemma 9 (see [26])**

Let *ω* ∈ *A*_*∞*_ and *b* ∈ *BMO*(*R* ^*n*^). Then, for any cube *Q* ⊂ *R* ^*n*^,

(33) $$\begin{array}{c} \frac{1}{\omega \left(Q\right)}\int_{Q}\exp\left(\frac{\left|b\left(x\right)-b_{Q}\right|}{C_{0}{||b||}_{*}}\right)\omega \left(x\right)dx\leq C,\\ {||b-b_{Q}||}_{\exp L,\;Q,\;\omega }\leq C{||b||}_{*}, \end{array}$$

where *C*_0_ and *C* are positive constants independent of *b* and *Q*, and ||*b*||_∗_ is the *BMO*-norm of *b*.

**Lemma 10 (see [28])**

Let 1 ≤ *p* < *∞*, *ω* ^*p*^ ∈ *A*_1_, *b*_*j*_ ∈ *BMO*(*R* ^*n*^)  (*j* = 1,…, *m*), and *Q* be a cube. Then for any positive integer *m* and *k* = 0,1,…,

(34) (1|2kQ|∫2kQωp(x)∏j=1m|bj(x)−(bj)Q|pdx)1/p  ≤C||b→||∗(k+1)m
ess
 inf⁡y∈Q⁡ω(y).  

## 3. Proof of Theorem 6: The Case *m*  =  1

When *m* = 1, we write *b* = *b*_1_ and $T_{b}=T_{\vec{b}}$ for simplicity. We need to prove that, for *ω* ∈ *A*_1_ and *b* ∈ *BMO*(*R* ^*n*^), there exists constant *C* > 0 such that, for all *λ* > 0,

(35) ω({x∈Rn:|Tbf(x)|>λ})  ≤C∫Rn|f(y)|λ(1+log⁡+|f(y)|λ)ω(y)dy.

For any fixed *λ* > 0, we consider the Calderón-Zygmund decomposition of *f* at height *λ* and get a sequence of nonoverlapping cubes {*Q*_*i*_}, where *Q*_*i*_ = *Q*(*y*_*i*_, *r*_*i*_) is a cube centered at *y*_*i*_ with radius *r*_*i*_, such that

(36) $$\begin{array}{c} \left|f\left(x\right)\right|\leq \lambda ,\;\text{for}\;\;\text{a}.\text{e}.\;\;x\in R^{n}\setminus \cup_{i}Q_{i}, \end{array}$$

(37) $$\begin{array}{c} \lambda <\frac{1}{\left|Q_{i}\right|}\int_{Q_{i}}\left|f\left(x\right)\right|dx\leq 2^{n}\lambda ,\;i=1,2,\ldots . \end{array}$$

Denote by *f*|_*Q*_*i*__ the restriction of *f* to *Q*_*i*_. Let *g*_*i*_(*x*) be the projection of *f*|_*Q*_*i*__ onto *Y*_*i*_ = span⁡{*ϕ*_1_(·−*y*_*i*_),  *ϕ*_2_(·−*y*_*i*_),…, *ϕ*_*l*_(·−*y*_*i*_)}. We decompose *f* into two parts, *f* = *g* + *h*, where

(38) $$\begin{array}{c} g\left(x\right)=\{\begin{array}{cc} f\left(x\right), & x\in R^{n}\setminus \cup_{i}Q_{i},\\ g_{i}\left(x\right), & x\in Q_{i},\;\;i=1,2,\ldots , \end{array} \end{array}$$

and *h*(*x*) = *f*(*x*) − *g*(*x*) = ∑_*i*_ *h*_*i*_(*x*) with *h*_*i*_(*x*) = *f*(*x*) − *g*_*i*_(*x*) for *x* ∈ *Q*_*i*_.

Obviously, *h*_*i*_ is supported on *Q*_*i*_ and it follows from (18) that, for any 1 ≤ *k* ≤ *l* and any *i* (also see [2] p.170 or [3] (3.13)),

(39) $$\begin{array}{c} \int_{Q_{i}}\phi_{k}\left(x-y_{i}\right)h_{i}\left(x\right)dx=0. \end{array}$$

Furthermore, we have

(40) $$\begin{array}{c} \left|g\left(x\right)\right|\leq C\lambda ,\;\text{a}.\text{e}.\;x\in R^{n}. \end{array}$$

Indeed, by (36) and (38) we have |*g*(*x*)|≤*λ*, for a.e. *x* ∈ *R* ^*n*^∖∪_*i*_ *Q*_*i*_. On the other hand, for any *x* ∈ ∪_*i*_ *Q*_*i*_ there exists an *i* so that *x* ∈ *Q*_*i*_, and noting that *g*_*i*_(*x*) is the projection of *f*|_*Q*_*i*__ onto *Y*_*i*_, then it follows from Lemma 7 and (37) that

(41) |g(x)|=|gi(x)|≤sup⁡y∈Qi|gi(y)|≤C|Qi|∫Qi|f(y)|dy≤Cλ.

So, (40) is verified.

Since *ω* ∈ *A*_1_, then by (38), (41), and (16), we have

(42) ∫Rn|g(x)|ω(x)dx ≤∫Rn∖∪iQi|f(x)|ω(x)dx+∫∪iQi|gi(x)|ω(x)dx ≤∫Rn|f(x)|ω(x)dx  +∑i∫Qi(C|Qi|∫Qi|f(y)|dy)ω(x)dx ≤∫Rn|f(x)|ω(x)dx+C∑iω(Qi)|Qi|∫Qi|f(y)|dy ≤C∫Rn|f(x)|ω(x)dx  +C∑i∫Qi|f(y)|(ess inf⁡x∈Qi⁡ω(x))dy ≤C∫Rn|f(x)|ω(x)dx.

For any cube *Q*_*i*_, by (16) and (37) we have

(43) ω(Qi)≤C|Qi|ess inf⁡y∈Qi⁡ω(y)≤Cλ−1∫Qi|f(x)|(ess inf⁡y∈Qi⁡ω(y))dx≤Cλ−1∫Qi|f(x)|ω(x)dx.  

Set $Q_{i}^{*}=2\sqrt{n}Q_{i}$ and *Ω* = ∪_*i*_ *Q*_i_*; then

(44) $$\begin{array}{c} \omega \left(\Omega \right)\leq \underset{i}{\sum }\omega \left(Q_{i}^{*}\right)\leq C\underset{i}{\sum }\omega \left(Q_{i}\right)\leq C\lambda^{-1}{||f||}_{L^{1}(\omega )}. \end{array}$$

Thus

(45) ω({x∈Rn:|Tbf(x)|>λ}) ≤ω({x∈Rn∖Ω:|Tbf(x)|>λ2})+ω(Ω) ≤ω({x∈Rn∖Ω:|Tbg(x)|>λ2})  +ω({x∈Rn∖Ω:|Tbh(x)|>λ2})  +Cλ−1||f||L1(ω) =I+J+Cλ−1||f||L1(ω).

For any *p* > 1, since *ω* ∈ *A*_1_ ⊂ *A*_*p*_, then by Theorem 5, (40), and (42), we have

(46) I≤Cλ−p∫Rn|Tbg(x)|pω(x)dx≤Cλ−p∫Rn|g(x)|pω(x)dx≤Cλ−1∫Rn|g(x)|ω(x)dx≤Cλ−1∫Rn|f(x)|ω(x)dx.

For the second term *J*, since

(47) Tbh(x)=∑iTbhi(x)=∑i(b(x)−bQi)Thi(x)−∑iT((b(x)−bQi)hi)(x),

then

(48) J≤ω({x∈Rn∖Ω:|∑i(b(x)−bQi)Thi(x)|>λ4})+ω({x∈Rn∖Ω:|∑iT((b(x)−bQi)hi)(x)|>λ4})=J(1)+J(2).

Let us consider *J* ^(1)^ first. Applying (39), condition (*K*_4_), and Lemma 10, we have

(49) J(1)≤Cλ∫Rn∖Ω|∑i(b(x)−bQi)Thi(x)|ω(x)dx≤Cλ∑i∫Rn∖Qi∗|b(x)−bQi|     ×|∫QiK(x−y)hi(y)dy|ω(x)dx≤Cλ∑i∫Rn∖Qi∗|b(x)−bQi|     ×(∫Qi|K(x−y)−∑k=1lBk(x−yi)ϕk(y−yi)|        ×|hi(y)|dy)ω(x)dx≤Cλ∑i∫Qi|hi(y)|     ×(∫Rn∖Qi∗|K(x−y)           −∑k=1lBk(x−yi)ϕk(y−yi)|        ×|b(x)−bQi|ω(x)dx)dy≤Cλ∑i∫Qi|hi(y)|(∫|x−yi|>2|y−yi||y−yi|γ|x−y|n+γ           ×|b(x)−bQi|ω(x)dx)dy≤Cλ∑i∫Qi|hi(y)|     ×(∑s=1∞∫2s|y−yi|<|x−yi|≤2s+1|y−yi||y−yi|γ|x−yi|n+γ         ×|b(x)−bQi|ω(x)dx)dy≤Cλ∑i∫Qi|hi(y)|(∑s=1∞12sγ(2s+1|y−yi|)n            ×∫2s+2Qi|b(x)−bQi|ω(x)dx)dy≤Cλ∑i∫Qi|hi(y)|(∑s=1∞12sγ||b||∗(s+3)ess inf⁡x∈Qi⁡ω(x))dy≤Cλ∑i∫Qi|hi(y)|ω(y)dy.

It follows from (42) that

(50) J(1)≤Cλ∫Rn(|f(y)|+|g(y)|)ω(y)dy≤Cλ∫Rn|f(y)|ω(y)dy.

Now, let us consider *J* ^(2)^. By the weak type (1,1) estimate of *T* (see Theorem 3), (27), (41), and Lemmas 9 and 10, we have

(51) J(2)≤ω({x∈Rn:|T(∑i(b−bQi)hi)(x)|>λ4})≤Cλ∫Rn|∑i(b(x)−bQi)hi(x)|ω(x)dx≤Cλ∑i∫Qi|b(x)−bQi||hi(x)|ω(x)dx≤Cλ∑i∫Qi|b(x)−bQi||f(x)−gi(x)|ω(x)dx≤Cλ∑i∫Qi|f(x)||b(x)−bQi|ω(x)dx +Cλ∑i∫Qi|gi(x)||b(x)−bQi|ω(x)dx≤Cλ∑iω(Qi)||f||Llog⁡L, Qi, ω||b−bQi||exp⁡L, Qi, ω +Cλ∑i∫Qi(1|Qi|∫Qi|f(y)|dy)|b(x)−bQi|ω(x)dx≤Cλ∑iω(Qi)||f||Llog⁡L, Qi, ω +Cλ∑i∫Qi|f(y)|(1|Qi|∫Qi|b(x)−bQi|ω(x)dx)dy≤Cλ∑iω(Qi)||f||Llog⁡L, Qi, ω +Cλ∑i∫Qi|f(y)|(ess inf⁡x∈Qi⁡ω(x))dy≤Cλ∑iω(Qi)||f||Llog⁡L, Qi, ω+Cλ∑i∫Qi|f(y)|ω(y)dy.

Note that (26) implies

(52) $$\begin{array}{c} {||f||}_{L\log L,\;Q_{i},\;\omega }\leq C\left\{\lambda +\frac{\lambda }{\omega \left(Q_{i}\right)}\int_{Q_{i}}\Phi \left(\frac{\left|f\left(y\right)\right|}{\lambda }\right)\omega \left(y\right)dy\right\}. \end{array}$$

Then by (43) we have

(53) J(2)≤C∑i{ω(Qi)+∫QiΦ(|f(y)|λ)ω(y)dy}+Cλ∫Rn|f(y)|ω(y)dy≤C∫RnΦ(|f(y)|λ)ω(y)dy+Cλ∫Rn|f(y)|ω(y)dy≤C∫Rn|f(x)|λ(1+log⁡+|f(y)|λ)ω(y)dy.  

Combining the estimates for *J* ^(1)^ and *J* ^(2)^, we have

(54) $$\begin{array}{c} J\leq C\int_{R^{n}}\frac{\left|f\left(x\right)\right|}{\lambda }\left(1+\log^{+}\frac{\left|f\left(y\right)\right|}{\lambda }\right)\omega \left(y\right)dy. \end{array}$$

This along with (45) and (46) gives (35), which is the desired result.

## 4. Proof of Theorem 6: The General Case *m* > 1

In this section, we will use an induction argument to prove Theorem 6 for the general case. To this end, we first introduce some notation.

As in [22], given positive integers *m* and *j*  (1 ≤ *j* ≤ *m*), we denote by *C*_*j*_^*m*^ the family of all finite subsets *σ* = {*σ*(1), *σ*(2),…, *σ*(*j*)} of {1,2,…, *m*} of *j* different elements. For any *σ* ∈ *C*_*j*_^*m*^, we write *σ*′ = {1,2,…, *m*}∖*σ*.

For $\vec{b}=(b_{1},\ldots ,b_{m})$ with *b*_*j*_ ∈ *BMO*(*R* ^*n*^) and *σ* = {*σ*(1), *σ*(2),…, *σ*(*j*)} ∈ *C*_*j*_^*m*^  (1 ≤ *j* ≤ *m*), we denote by ${\vec{b}}_{\sigma }=(b_{\sigma (1)},b_{\sigma (2)},\ldots ,b_{\sigma (j)})$, ${\vec{b}}_{\sigma^{\prime }}=(b_{\sigma^{\prime }(1)},\ldots ,b_{\sigma^{\prime }(m-j)})$, and ${||\vec{b}||}_{*}={||b_{1}||}_{*}\cdots {||b_{m}||}_{*}$, ${||{\vec{b}}_{\sigma }||}_{*}={||b_{\sigma (1)}||}_{*}\cdots {||b_{\sigma (j)}||}_{*}$. Write

(55) $$\begin{array}{c} {\left(\vec{b}\left(x\right)-\vec{b}\left(y\right)\right)}_{\sigma }=\overset{j}{\underset{i=1}{\prod }}\left(b_{\sigma (i)}\left(x\right)-b_{\sigma (i)}\left(y\right)\right),\\ {\left(\vec{b}\left(y\right)-{\vec{b}}_{Q}\right)}_{\sigma }=\overset{j}{\underset{i=1}{\prod }}\left(b_{\sigma (i)}\left(y\right)-{\left(b_{\sigma (i)}\right)}_{Q}\right), \end{array}$$

where *Q* is a cube in *R* ^*n*^ and ${\vec{b}}_{Q}=({\left(b_{1}\right)}_{Q},\ldots ,{\left(b_{m}\right)}_{Q})$. We also need the following notation:

(56) $$\begin{array}{c} T_{{\vec{b}}_{\sigma }}f\left(x\right)=\int_{R^{n}}{\left(\vec{b}\left(x\right)-\vec{b}\left(y\right)\right)}_{\sigma }K\left(x,y\right)f\left(y\right)dy. \end{array}$$

**Proof of Theorem 6 (the general case *m* > 1)**

We have proved that Theorem 6 is true for *m* = 1 in Section 3. Now, we assume that Theorem 6 holds for all positive integer *j* < *m*; namely, for all 1 ≤ *j* < *m* and any *σ* ∈ *C*_*j*_^*m*^, we have

(57) $$\begin{array}{c} \omega \left(\left\{x\in R^{n}:\left|T_{{\vec{b}}_{\sigma }}f\left(x\right)\right|>\lambda \right\}\right)\leq C\int_{R^{n}}\Phi_{j}\left(\frac{\left|f\left(y\right)\right|}{\lambda }\right)\omega \left(y\right)dy. \end{array}$$

For any fixed *λ* > 0, we consider the Calderón-Zygmund decomposition of *f* at height *λ* as in Section 3 and use the notations {*Q*_*i*_}, *Q*_i_*, *g*, *h*, *h*_*i*_, and *Ω* as there.

For the same reason as in (45), we have

(58) ω({x∈Rn:|Tb→f(x)|>λ})  ≤ω({x∈Rn∖Ω:|Tb→f(x)|>λ})+ω(Ω)  ≤ω({x∈Rn∖Ω:|Tb→g(x)|>λ2})   +ω({x∈Rn∖Ω:|Tb→h(x)|>λ2})   +Cλ−1||f||L1(ω)  :=I+J+Cλ−1||f||L1(ω).

Similar to (46), we have

(59) I≤Cλ−p∫Rn|Tb→g(x)|pω(x)dx≤Cλ−p∫Rn|g(x)|pω(x)dx≤Cλ−1||f||L1(ω).

Then

(60) $$\begin{array}{c} \omega \left(\left\{x\in R^{n}:\left|T_{\vec{b}}f\left(x\right)\right|>\lambda \right\}\right)\leq J+C\lambda^{-1}{||f||}_{L^{1}(\omega )}. \end{array}$$

Reasoning as the proof of Lemma 3.1 in [22] (pp. 683-684), we have

(61) Tb→hi(x)=(b1(x)−(b1)Qi)⋯(bm(x)−(bm)Qi)Thi(x)+(−1)mT((b1−(b1)Qi)⋯(bm−(bm)Qi)hi)(x)+∑j=1m−1∑σ∈Cjm(−1)m−j∫Rn(b→(x)−b→Qi)σ(b→(y)−b→Qi)σ′           ×K(x,y)hi(y)dy.

Note that

(62) (b→(x)−b→Qi)σ =∏s=1j[(bσ(s)(x)−bσ(s)(y))+(bσ(s)(y)−(bσ(s))Qi)],(b→(y)−b→Qi)σ′=∏s=1m−j[bσ′(s)(y)−(bσ′(s))Qi],

and expanding ${\left(\vec{b}(x)-{\vec{b}}_{Q_{i}}\right)}_{\sigma }{\left(\vec{b}(y)-{\vec{b}}_{Q_{i}}\right)}_{\sigma^{\prime }}$, it is not difficult to check that

(63) Tb→hi(x)=(b1(x)−(b1)Qi)⋯(bm(x)−(bm)Qi)Thi(x)+CmT((b1−(b1)Qi)⋯(bm−(bm)Qi)hi)(x)+∑j=1m−1∑σ∈CjmCm,jTb→σ((b→−b→Qi)σ′hi)(x).

This gives

(64) |Tb→h(x)|=|∑iTb→hi(x)|≤∑i[∏j=1m|bj(x)−(bj)Qi|]|Thi(x)|+C|T(∑i[∏j=1m(bj−(bj)Qi)]hi)(x)|+C∑j=1m−1∑σ∈Cjm|Tb→σ(∑i(b→−b→Qi)σ′hi)(x)|.

Thus,

(65) J=ω({x∈Rn∖Ω:|Tb→h(x)|>λ2})≤ω({x∈Rn∖Ω:∑i[∏j=1m|bj(x)−(bj)Qi|]     ×|Thi(x)|>λ6}) +ω({x∈Rn∖Ω:C     ×|T(∑i[∏j=1m(bj−(bj)Qi)]hi)(x)|>λ6}) +ω({x∈Rn∖Ω:C     ×∑j=1m−1∑σ∈Cjm|Tb→σ(∑i(b→−b→Qi)σ′hi)(x)|>λ6}):=J1+J2+J3.

Applying (39), condition (*K*_4_), and Lemma 10, similar to the estimate of *J* ^(1)^ in Section 3, we have

(66) J1≤Cλ∑i∫Rn∖Ω[∏j=1m|bj(x)−(bj)Qi|]|Thi(x)|ω(x)dx≤Cλ∑i∫Rn∖Qi∗[∏j=1m|bj(x)−(bj)Qi|]     ×{∫Qi|K(x−y)−∑k=1lBk(x−yi)ϕk(y−yi)|       ×|hi(y)|dy}ω(x)dx≤Cλ∑i∫Qi|hi(y)|{∫|x−yi|>2|y−yi||y−yi|γ|x−y|n+γ           ×∏j=1m|bj(x)−(bj)Qi|ω(x)dx}dy≤Cλ∑i∫Qi|hi(y)|∑s=1∞{∫2s|y−yi|<|x−yi|≤2s+1|y−yi||y−yi|γ|x−yi|n+γ            ×∏j=1m|bj(x)−(bj)Qi|ω(x)dx}dy≤Cλ∑i∫Qi|hi(y)|∑s=1∞12sγ1(2s+1|y−yi|)n           ×∫2s+2Qi∏j=1m|bj(x)−(bj)Qi|            ×ω(x)dx dy≤Cλ∑i∫Qi|hi(y)|∑s=1∞(s+3)m2sγ||b→||∗ess inf⁡y∈Qi⁡ω(y)dy≤Cλ||f||L1(ω).

For *J*_2_, by the weak type (1,1) estimate for *T* (see Theorem 3), (27), (41), and Lemmas 9 and 10, similar to the estimate of *J* ^(2)^ in Section 3, we have

(67) J2≤Cλ∫Rn∑i|hi(x)|∏j=1m|bj(x)−(bj)Qi|ω(x)dx≤Cλ∑i∫Qi|f(x)|∏j=1m|bj(x)−(bj)Qi|ω(x)dx+Cλ∑i∫Qi|gi(x)|∏j=1m|bj(x)−(bj)Qi|ω(x)dx≤Cλ∑iω(Qi)||f||L(log⁡L)m, Qi, ω∏j=1m||bj−(bj)Qi||exp⁡L, Qi, ω+Cλ∑i∫Qi(1|Qi|∫Qi|f(y)|dy)     ×∏j=1m|bj(x)−(bj)Qi|ω(x)dx≤Cλ∑iω(Qi)||f||L(log⁡L)m, Qi, ω+Cλ∑i∫Qi|f(y)|    ×(1|Qi|∫Qi∏j=1m|bj(x)−(bj)Qi|ω(x)dx)dy≤Cλ∑iω(Qi)||f||L(log⁡L)m, Qi, ω+Cλ∑i∫Qi|f(y)|(ess inf⁡y∈Qi⁡ω(y))dy≤Cλ∑iω(Qi)||f||L(log⁡L)m, Qi, ω+Cλ∑i∫Qi|f(y)|ω(y)dy.

Then by (26) and (43) we have

(68) J2≤C∑i{ω(Qi)+∫QiΦm(|f(y)|λ)ω(y)dy}+Cλ∫Rn|f(y)|ω(y)dy≤C∫RnΦm(|f(y)|λ)ω(y)dy+Cλ∫Rn|f(y)|ω(y)dy≤C∫Rn|f(x)|λ(1+log⁡+|f(y)|λ)mω(y)dy.

Now, let us consider *J*_3_ by applying the induction hypothesis.

Noting that *h*_*i*_(*x*) = (*f*(*x*) − *g*_*i*_(*x*))*χ*_*Q*_*i*__(*x*)  (*i* = 1,2,…), we can split *J*_3_ into two parts

(69) J3≤ω({x∈Rn∖E:C    ×∑j=1m−1∑σ∈Cjm|Tb→σ(∑i(b→−b→Qi)σ′fχQi)(x)|    >λ12}) +ω({x∈Rn∖E:C     ×∑j=1m−1∑σ∈Cjm|Tb→σ(∑i(b→−b→Qi)σ′giχQi)(x)|     >λ12}):=J3(1)+J3(2).

For *σ* ∈ *C*_*j*_^*m*^, we denote by *σ*′ = {*σ*′(1), *σ*′(2),…*σ*′(*m* − *j*)}, so that

(70) $$\begin{array}{c} \left|{\left(\vec{b}-{\vec{b}}_{Q_{i}}\right)}_{\sigma^{\prime }}\right|=\left|b_{\sigma^{\prime }(1)}-{\left(b_{\sigma^{\prime }(1)}\right)}_{Q_{i}}\right|\cdots \left|b_{\sigma^{\prime }(m-j)}-{\left(b_{\sigma^{\prime }(m-j)}\right)}_{Q_{i}}\right|. \end{array}$$

From Lemma 9, there exist constants *C*_*s*,0_ and *C*_*s*_ such that for *s* = 1 ⋯ *m* − *j*

(71) $$\begin{array}{c} \frac{1}{\omega \left(Q_{i}\right)}\int_{Q_{i}}\exp\left(\frac{\left|b_{\sigma^{\prime }(s)}\left(x\right)-{\left(b_{\sigma^{\prime }(s)}\right)}_{Q_{i}}\right|}{C_{s,0}{||b_{\sigma^{\prime }(s)}||}_{*}}\right)\omega \left(x\right)dx\leq C_{s}. \end{array}$$

Set *γ*_*s*_ = (*C*_*s*,0_||*b*_*σ*′(*s*)_||_∗_)^−1^  (*s* = 1,…*m* − *j*); then it follows from the induction hypothesis and (31) that

(72) J3(1)≤C∑j=1m−1∑σ∈Cjm∫RnΦj(|f(y)|λ          ×∑i|(b→−b→Qi)σ′|χQi(y))ω(y)dy≤C∑j=1m−1∑σ∈Cjm∑i∫QiΦj(|f(y)|λ|(b→−b→Qi)σ′|)ω(y)dy≤C∑j=1m−1∑σ∈Cjm∑i∫QiΦm(|f(y)|γ1⋯γm−j·λ)ω(y)dy+C∑j=1m−1∑σ∈Cjm∑i{∑s=1m−j∫QiΨ(γs|bσ′(s)−(bσ′(s))Qi|)         ×ω(y)dy}.

By (71) and (43), we have

(73) ∑i{∑s=1m−j∫QiΨ(γs|bσ′(s)−(bσ′(s))Qi|)ω(y)dy} =∑i{∑s=1m−j∫Qi[exp⁡(|bσ′(s)(x)−(bσ′(s))Qi|Cl,0||b||∗)−1]        ×ω(y)dy} ≤∑i∑s=1m−jCsω(Qi) ≤Cλ−1∫Rn|f(y)|ω(y)dy.

Noting that Φ_*m*_(*ab*) ≤ *C*Φ_*m*_(*a*)Φ_*m*_(*b*) for *a*, *b* > 0, we have

(74) J3(1)≤C∑j=1m−1∑σ∈Cjm∫RnΦm(|f(y)|λ)      ×Φm(1γ1⋯γm−j)ω(y)dy+Cλ−1∫Rn|f(y)|ω(y)dy≤C∫RnΦm(|f(y)|λ)ω(y)dy.

Finally, we consider *J*_3_^(2)^. By Jensen's inequality,

(75) Φm(|fQi|λ)≤Φm(1|Qi|∫Qi|f(x)|λdx)≤1|Qi|∫QiΦm(|f(x)|λ)dx.

By the induction hypothesis, (31), and (75), similar to the estimate of *J*_3_^(1)^, we have

(76) J3(2)≤C∑j=1m−1∑σ∈Cjm∫RnΦj(|fQi|λ∑i|(b→−b→Qi)σ′|χQi(y))     ×ω(y)dy≤C∑j=1m−1∑σ∈Cjm∑i∫QiΦj(|fQi|λ|(b→−b→Qi)σ′|)ω(y)dy≤C∑j=1m−1∑σ∈Cjm∑i∫QiΦm(|fQi|γ1⋯γm−j·λ)ω(y)dy+C∑j=1m−1∑σ∈Cjm∑i{∑s=1m−j∫QiΨ(γs|bσ′(s)−(bσ′(s))Qi|)           ×ω(y)dy}≤C∑j=1m−1∑σ∈Cjm∑i∫Qi{1|Qi|∫QiΦm(|f(x)|λ)dx}ω(y)dy+Cλ−1∫Rn|f(y)|ω(y)dy.

Applying (16), we have

(77) ∫Qi{1|Qi|∫QiΦm(|f(x)|λ)dx}ω(y)dy =∫QiΦm(|f(x)|λ){1|Qi|∫Qiω(y)dy}dx ≤∫QiΦm(|f(x)|λ)ess inf⁡y∈Qi⁡ω(y)dx ≤∫QiΦm(|f(x)|λ)ω(x)dx.

Then,

(78) J3(2)≤C∑j=1m−1∑σ∈Cjm∑i∫QiΦm(|f(x)|λ)ω(x)dx+Cλ−1∫Rn|f(y)|ω(y)dy≤C∫RnΦm(|f(y)|λ)dy.

This along with (69) and (74) gives

(79) $$\begin{array}{c} J_{3}\leq C\int_{R^{n}}\Phi_{m}\left(\frac{\left|f\left(x\right)\right|}{\lambda }\right)dx. \end{array}$$

By (60), (65), and the above estimates for *J*_1_, *J*_2_, and *J*_3_, we obtain

(80) ω({x∈Rn:|Tb→f(x)|>λ}) ≤C∫RnΦm(|f(x)|λ)dx+Cλ−1||f||L1(ω) ≤C∫Rn|f(y)|λ(1+log⁡+|f(y)|λ)mω(y)dy.

The proof of the general case of Theorem 6 is therefore completed.
